# Slow Poisoning by Preparations of Lead, and Its Influence on the Product of Conception

**Published:** 1861-04

**Authors:** Constantine Paul


### SLOW POISONING BY PREPARATIONS OF LEAD,

### AND ITS INFLUENCE ON THE PRODUCT OF CONCEPTION.

BY CONSTANTINE PAUL,

(*Arch. Gen. de Med., May,* 1860.)

M. Paul, an *interne* of the Paris hospital, has drawn up a valuable memoir on the effects of lead-poisoning upon the product of conception. We will relate one of his observations as an example, and present a summary of his researches. In February, 1859, a woman entered the Necker Hospital, who had been for eight years working as a polisher of printing type. She was suffering from metrorrhagia, and had an evident saturnine cachexia. She had enjoyed good health, and had been delivered of three children, happily before taking to the occupation of polisher. Since then her health had been much shattered by lead-diseases. Three months after entering upon this trade she had a first attack of colic, and four years later another. At this time she became pregnant, and bore a dead child. Three years later still, she bore a child which died at the age of five months. She had eight pregnancies all terminating in abortion at two or three months, attended with excessive metrorrhagia. She recovered in M. Bouley's ward, under tonic and restorative treatment.

This case led M. Paul to extended inquiries in the typefoundries and elsewhere. He found that those women almost alone who handle the type are affected by saturnine diseases. In a first series of observations, he found that 4 women had had 15 ascertained pregnancies—of these, 10 ended in abortion, 2 in premature labor, 1 in still-birth, and 1 child died within twenty-four hours.

In a second series of cases, 5 women had borne an aggregate of 9 children at term before exposing themselves to lead, and had no abortion or accident of pregnancy. Since exposure to lead they had 36 pregnancies; of these, 26 ended in abortion at from two to six months; 1 in premature labor; 2 in still-birth; 5 children died, 4 of which within the first year; and 2 children were living, 1 being puny and ailing, the other only three years old.

In a third order, a woman had during her employment in a type foundry five pregnancies, all ending in abortion. She quitted the business and bore a healthy child.

In a fourth order, is the case of a woman who, having left the trade for two periods, bore during these intervals of freedom two healthy children; returning to the trade had two abortions.

In a fifth series M. Paul shows that the same disastrous influence is felt when the fathers handle lead. In 7 cases, every woman had an abortion; of 32 pregnancies occurring during the husband's exposure to lead, 12 children were born prematurely. Of twenty living children, 8 died in the first year, 3 in the second, 5 in the third, 1 after the third year, 2 remained living.

In the sixth series the author shows that where the lead affection was less marked there was a corresponding diminution of the injurious effect upon the product of conception.—*S. M. & S. Journal.*

